# Distant metastasis detected by routine staging in breast cancer patients participating in the national German screening programme: consequences for clinical practice

**DOI:** 10.1186/s40064-016-2703-6

**Published:** 2016-07-07

**Authors:** Peter Rusch, Oliver Hoffmann, Anna-L. Stickelmann, Stephan Böhmer, Regine Gätje, Karl G. Krüger, Stefan Niesert, Andrea Schmidt, Rainer Kimmig

**Affiliations:** Universitätsfrauenklinik Essen, Hufelandstr. 55, 45122 Essen, Germany; Elisabeth-Krankenhaus Essen, Klara-Kopp-Weg 1, 45138 Essen, Germany; Evangelisches Krankenhaus Oberhausen, Virchowstr. 20, 46047 Oberhausen, Germany; Alfried Krupp Krankenhaus, Alfried-Krupp-Straße 21, 45131 Essen, Germany; Evangelisches Krankenhaus Mülheim, Wertgasse 30, 45468 Mülheim an der Ruhr, Germany; Diavero Diagnosezentrum Essen, Heidbergweg 22, 45257 Essen, Germany

**Keywords:** Routine staging, Breast cancer, Screening, Distant metastases

## Abstract

**Purpose:**

To determine frequency of routine radiological staging of breast cancer patients diagnosed in a German Breast Cancer Screening Center from 2007 to 2014, the incidence and consequences of distant metastases detected and the resulting implications for clinical routine.

**Methods:**

Records of 896 patients with primary breast cancer diagnosed in the Screening Centre and treated in five participating hospitals were analyzed retrospectively. Evaluation included frequency and type of staging procedures and results with respect to distant metastasis and their consequences on clinical management.

**Results:**

894/896 Patients (99.8 %) received staging for distant metastases by bone scintigraphy, chest X-ray and liver sonography and/or CT/MRT diagnostics. Distant metastasis was suggested In 6/894 patients but excluded in 3 by further diagnostics or clinical course. Thus, 3 (0.3 %) were clinically verified to have metastatic disease in bone (n = 2; both pT2) or in bone and lung (n = 1; cT4, cN3).

**Conclusion:**

Due to the low incidence of verified metastatic disease, the high false positive rate of staging procedures and the unfavorable cost/benefit ratio routine radiological staging should be completely omitted in asymptomatic breast cancer patients diagnosed in a breast cancer screening programme.

## Background

Breast cancer is the most frequent malignant disease in women in Europe; in Germany the incidence is 70,000 new breast cancer diagnoses annually (Kaatsch et al. 2014). Aiming at an improved breast-cancer-therapy several countries introduced screening-programs for early breast cancer detection by mammography (Giordano et al. 2012; Shapiro et al. 1998). In Germany a suchlike program started in accordance with European guidelines in 2004, since 2009 a region wide maintenance exists in all 16 states, organized in more than 94 so-called Breast-Cancer-Screening-Units. The German Breast-Cancer-Screening-Programme invites breast-healthy women of 50–69 years of age in a 2-year-interval by the local residents` registration offices. After diagnosis of breast cancer via Screening-Programme further oncological treatment follows national and international guidelines. Although a routine radiological staging for exclusion of distant metastasis in early breast cancer is not recommended by current guidelines (Kreienberg et al. 2013; Gradishar et al. 2016), it seems still to be performed in the majority of patients (Chand et al. 2013; Simos et al. 2015). Modalities focus on organs which would be primarily involved: the skeletal system via whole-body-scintigraphy, the lung via chest-X-ray and the liver via ultrasound and/or CT- or MRI-scan in some cases. Thus, in this study we analyzed the frequency of radiological routine staging in breast cancer patients diagnosed in a German Breast-Cancer-Screening-Center from 2007 to 2014, the findings and their consequences on further treatment. The results should give insight into the clinical routine management in the region investigated and will be discussed with respect to side effects, e.g. cost/benefit-ratio.

## Methods

896 Patients who were diagnosed with primary breast cancer in the German Breast Cancer Screening Center Diavero^®^/Essen between 2007 and 2014 via the German Mammography-Screening-Programme were included in the study. For these patients the records of Diavero^®^ and the five in the study participating hospitals have been analyzed with respect to type and results of staging procedures to preclude distant metastasis and whether these results changed the oncological treatment plan. All research presented in this paper was conducted in accordance with the ethical research standards of the local ethics committee.

**Fig. 1:**
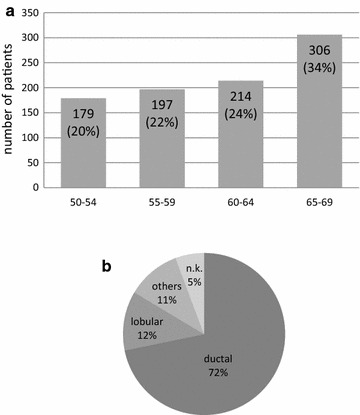
**a** Age-distribution. **b** Histological type

The characteristics of the patients and the initial tumor stages are summarized in Fig. 1 and Tables 1 and 2 (T = tumor size; N = nodal involvement; n.k. = not known).

**Table 1:** Prior mammography in medical history

	National screening programme			Other indication	None
	2 year before	4 year before	>4 year before	>1 year before	
n	249	210	26	274	137
%	28.1	23.1	3.1	30.6	15.1

**Table 2:** T-, N-Status, Grading

T-Status	n	%	N-status	n	%	Grading	n	%
1	689	76.9	N 0	696	77.7	1	213	23.8
2	180	20.1	N 1	142	15.8	2	485	54.1
3	16	1.8	N 2	35	3.9	3	154	17.2
4	5	0.5	N 3	14	1.6	n.k.	44	4.9
n.k.	6	0.7	n.k.	9	1.0			

Patients with detected early breast cancer in the National Screening Programme at Diavero^®^ were sent to treating hospitals of their choice afterwards. This work evaluates all patients referred to one of the five hospitals participating in this study. Staging procedures for detection of metastases were conducted after hospitals own standards. Decision on oncological treatment was made in local interdisciplinary tumor board and submitted as proposal to the patient.

## Results

### Incidence, modality and timing of staging

894/896 (99.8 %) of the patients received a routine staging for distant metastasis. In 618 (69 %) detailed information about the modality of staging was known: 340 Patients had the “classical approach” of a whole-body-scintigraphy, a chest-X-ray and a liver-ultrasound, but in 33 cases use of an additional modality like CT, PET-CT or MRI to exclude metastases was documented. On the other hand 278 cases got a non-conventional staging by CT scan as a substitute for X-ray or ultrasound, but still in 3 cases an additional CT, PET-CT or MRI was necessary to exclude metastases.

In 276 cases the staging result of bone, lung and liver is known—but no report was available to verify the staging modality used. In 2 patients only a staging was omitted, so they were assigned to MX-Status.

In 236 cases timing of staging with respect to the start of oncological treatment could be evaluated: 198 patients (84 %) had a complete staging preoperatively, n = 38 postoperatively (16 %).

### Results of staging (M0, M1, MX)

In 6 of 894 patients with radiological staging metastatic spread to the skeletal system (and lung in one case) at point of primary breast cancer diagnosis has been suggested. In three of these evidence for metastasis in imaging was highly conclusive, so these patients were assigned to palliative treatment without histological confirmation. One of them, however, was diagnosed with a stage cT4-tumor, thus in fact she did not fulfill criteria for participation in German Mammography Screening, which aims at woman without clinical signs of breast cancer.

In a 4th case the patient reported a traumatic event in medical history as a differential diagnosis for bone metastasis. This patient was assigned to adjuvant treatment. Follow-ups did not confirm distant metastasis and this patient remained in adjuvant treatment concept.

A 5th patient had the initial staging suspicion of bone-metastasis but treatment choice remained adjuvant. Follow-ups by whole-body-scintigraphy and MR-scan finally excluded bone metastases.

In a 6th patient the suspicion of distant bone metastasis was excluded via biopsy. The histological result confirmed the patients hyperparathyreoidism as the cause for multiple osteolytic lesions in imaging. The patient was finally staged M0, treatment followed adjuvant criteria.

### Implications for oncological treatment

The oncological concept has been modified in 3/894 patients due to the findings in staging for distant disease. Characteristics of the patients and treatment chosen are reported.

#### Patient 1

The 68 year old patient had bowel-cancer in medical history 30 years ago. She was first time participating in German Breast Cancer Screening Programme. She presented with a screening-detected hormone-receptor-positive ductal invasive breast cancer, G2, Ki67 = 20 %. Mastectomy was done at the patients request with sentinel-node axillary staging. The histological result was pT2 pN0 (sn-) L0 R0. The postoperative staging by whole-body-scintigraphy showed suspicion of bone metastasis, which was confirmed by MR- and CT-scan. Due to imaging—but without histological confirmation—the patient was assigned to palliative endocrine therapy and bone-modifying RANKL-antibody (Denosumab) and additional radiotherapy of pelvic bone metastasis.

#### Patient 2

In the 59 year old patient a hormone-receptor-positive ductal invasive breast cancer was diagnosed. Breast conserving therapy and axillary staging resulted in pT2 pN1a G3 ER90 % PR90 % Her2neg breast cancer. Timing of staging was not known but resulted in suspicion of bone metastasis to the lumbar spine and was confirmed by MR-scan. The patient has been treated deviating from guidelines by 4 cycles of epirubicin/cyclophosphamide chemotherapy followed by aromatase inhibitors. Bisphosphonates and local radiotherapy of the lumbar spine were added.

#### Patient 3

The 58 year old patient was first time participating in Screening, but presented with an inflammatory cT4 breast cancer and clinical signs of axillary lymph node metastasis. Histology confirmed a ductal invasive type of breast cancer, hormone-receptor-positive, Her2-negative, no Ki67-value was documented. Staging showed distant metastasis in bone and lung which lead to palliative chemotherapy with Paclitaxel and anti-VEGF-antibody Bevacizumab.

Patients 4–6, whose metastasis were not confirmed, were between 60 and 65 years old. All of them showed invasive ductal breast cancer, pT1c, pN0 (sn-), G2, R0. Metastasis could be precluded by follow up control in 2 patients, and by biopsy in 1 patient, which revealed a hyperparathyroidism. All were treated in the adjuvant protocol according to the current guidelines.

## Discussion

International and also the German Breast Cancer-Screening-Programme by mammography aim at improving cure for breast-cancer with respect to breast cancer specific mortality and overall survival concomitantly reducing patients burden by unnecessary diagnostics or treatment due to early diagnosis (Waters and Porter 2014; Australian Institute of Health and Welfare 2016).

However, with respect to different treatment protocols in the adjuvant and palliative setting it is important to preclude metastatic disease in patients diagnosed with breast cancer. This leads to routine clinical diagnostics in all breast cancer patients for the detection of lung, liver and bone metastases (Chand et al. 2013; Simos et al. 2015; Puglisi et al. 2005; Rayson and Porter 2015; Myers et al. 2001). On the other hand, incidence of distant metastasis in early breast cancer is low (Puglisi et al. 2005; Barrett et al. 2009; Norum and Andreassen 2000) and staging is accompanied by a high percentage of false positives (Myers et al. 2001; Norum and Andreassen 2000; Nomura et al. 1978), therefore, routine staging for metastasis in asymptomatic early breast cancer is no longer recommended in a variety of national and international guidelines (Kreienberg et al. 2013; Gradishar et al. 2016; Brennan and Houssami 2012; Ravaioli et al. 2002).

Although, the first recommendations not to stage asymptomatic patients with early breast cancer have been published almost 40 years ago (Nomura et al. 1978; Butzelaar et al. 1977), this seems not to be generally implemented in clinical routine up to now (Simos et al. 2015; Waters and Porter 2014; Simos and Clemons 2014). Chand et al. (Chand et al. 2013) could show that in the UK neither the indication nor the staging modality has been uniformly agreed upon by the UK Breast Surgeons. Same has been reported for Canada (Simos and Clemons 2014). In Germany, staging for distant metastases in early breast cancer also still seems to be widely spread at present in spite of the low detection rate (Debald et al. 2014).

Since breast cancer patients diagnosed in a national screening programme usually suffer from early asymptomatic disease, analysis of frequency of radiological staging, incidence and consequences of distant metastases may give randomly insight in current clinical routine. The result of 99.8 % patients staged for distant metastases in our region impressively confirmed the nonconformity of current clinical practice to scientific findings and resulting clinical guidelines. Even after the publication of the “Top Five List for Oncology” of ASCO in 2012 (Schnipper et al. 2012) no change was observed.

The diagnosis of distant metastases (bone) has been made in 2/896 (0.2 %) asymptomatic patients and no liver or lung metastases were observed (the patient with cT4 tumour had been excluded due to symptomatic disease). This extremely low rate of metastases corresponds well to that reported for stage I and II breast cancer in the UK (Barrett et al. 2009). The diagnosis of bone metastases in the two patients lead to changes in treatment protocol. However, there is no evidence that this change has any impact on survival; on the other hand toxicity of systemic treatment has been reduced at least in one patient. This, however, has to be balanced against the strain of three patients with false positive findings, who might have suffered from additional diagnostics and the psychological burden of diagnosis of incurable disease and its sequelae.

Apparently, approximately 3000 diagnostic staging procedures were performed unnecessarily. 894/896 patients of this cohort had to tolerate diagnostic procedures without any benefit. In principal, each exposure with ionizing radiation increases the risk of mutation and insofar incidence of cancer (Berrington de González and Darby 2004); a conventional staging with bone scintigraphy and chest X-ray causes 1.5 times more radiation load than natural exposure (2.1 mSv) throughout the year (Federal Office for Radiation Protection 2015). While basic radiological diagnostics will at least contribute to accumulation of ionizing exposure during lifetime additional diagnostic procedures will even increase the risk (Berrington de González and Darby 2004; Huang et al. 2009): in our collective more than one third (34 %) of examinations was associated with an extra radiation burden by use of CT or PET-CT for initial or extended staging in unclear cases. So direct burden will not only result from ionizing exposure but also from the risk of false positive results (Myers et al. 2001; Barrett et al. 2009) implying incurable disease. Besides that patients are exposed to unnecessary fear and distress waiting for their final results (Flory and Lang 2011; Baqutayan and Mohamed 2012)—especially if the suspicion of metastasis remains unsolved.

Economically, cost for staging procedures vary between countries; Morris et al. calculated cost of € 50,850/per case diagnosed with bone metastases in a center in Ireland (Morris et al. 2009). In Germany—depending on the medical fee schedule—cost for bone scintigraphy, chest X-ray and liver sonography range between 100 and 170€ per case without any additional diagnostics. Additional CT or even PET-scan leads to costs more than twice as much (Kassenärztliche Bundesvereinigung 2016; 2008). With respect to the annually new breast cancer diagnoses of stage I and II in Germany (Eisemann et al. 2013) this alone would generate between 5 Mio € and 20 Mio €/year for unnecessary staging.

In conclusion, the chance to be free of detectable distant metastases in our cohort of patients diagnosed within the national breast cancer screening programme has been > 99.5 % (Brennan and Houssami 2012; Debald et al. 2014)and this correlates well with the results of other studies with respect to asymptomatic breast cancer stage I and II. These findings contrast to the observation that 99.8 % in fact received radiological routine staging for distant metastases. As outlined it is obvious that routine clinical staging for distant metastases under these conditions will only harm the patients and not benefit at all. Even for clinical studies it should be evaluated strictly whether staging for distant metastases in early breast cancer is justified with respect to the extremely low prevalence and to the potential damage in the individual setting. Independently of medical aspects, the money spent for unnecessary staging procedures will lack for other services of the health care systems.

Thus, there is no argument any longer justifying radiological staging in asymptomatic breast cancer patients stage I and II or diagnosed in a screening program, neither with respect to safety, nor to forensic or economic reasons. Staging under these conditions has to be omitted completely not only in guidelines but also in clinical practice.
